# Increased core body temperature in astronauts during long-duration space missions

**DOI:** 10.1038/s41598-017-15560-w

**Published:** 2017-11-23

**Authors:** Alexander C. Stahn, Andreas Werner, Oliver Opatz, Martina A. Maggioni, Mathias Steinach, Victoria Weller von Ahlefeld, Alan Moore, Brian E. Crucian, Scott M. Smith, Sara R. Zwart, Thomas Schlabs, Stefan Mendt, Tobias Trippel, Eberhard Koralewski, Jochim Koch, Alexander Choukèr, Günther Reitz, Peng Shang, Lothar Röcker, Karl A. Kirsch, Hanns-Christian Gunga

**Affiliations:** 1 Charité – Universitätsmedizin Berlin, corporate member of Freie Universität Berlin, Humboldt-Universität zu Berlin, and Berlin Institute of Health, Institute of Physiology, Center for Space Medicine and Extreme Environments, CharitéCrossOver (CCO), Charitéplatz 1, Berlin, 10117 Germany; 2 Division of Sleep and Chronobiology, Department of Psychiatry, Perelman School of Medicine at the University of Pennsylvania, 1019 Blockley Hall, 423 Guardian Drive, Philadelphia, PA 19104-6021 USA; 3 German Air Force, Centre of Aerospace Medicine, Aviation Physiology Training Centre, Aviation Physiology Diagnostics and Science, Steinborner Str. 43, 01936, Königsbrück, Germany; 4 Department of Biomedical Sciences for Health, Università degli Studi di Milano, via Luigi Mangiagalli 31, 20133 Milan, Italy; 5 Department of Health and Kinesiology, Lamar University, Beaumont, TX 77710 USA; 6 Biomedical Research and Environmental Sciences Division, NASA Johnson Space Center, Houston, TX 77058 USA; 7 Preventive Medicine and Community Health, University of Texas Medical Branch, 301 University Boulevard, Galveston, TX 77555 USA; 8 Charité Medizinische Klinik, Charité Universitätsmedizin Berlin, Kardiologie, Augustenburger Platz 1, Berlin, 13353 Germany; 9 Drägerwerk AG & Co. KGaA, Moislinger Allee 53-55, Lubeck, 23558 Germany; 10 Department of Anaesthesiology, Hospital of the University of Munich, Marchioninistrasse 15, München, 81377 Germany; 11 DLR, Institut für Luft- und Raumfahrtmedizin, Abteilung Strahlenbiologie, Linder Höhe, Köln, 51147 Germany; 12 Nuclear Physics Institute of the Czech Academy of Sciences, Department of Radiation Dosimetry, Na Truhlářce 39/64, Praha 8, 180 00 Czech Republic; 13 Key Laboratory for Space Bioscience & Biotechnology, Institute of Special Environnments Biophysics, School of Life Sciences, Northwestern Polytechnical University, Xi’an, 710072 China

## Abstract

Humans’ core body temperature (CBT) is strictly controlled within a narrow range. Various studies dealt with the impact of physical activity, clothing, and environmental factors on CBT regulation under terrestrial conditions. However, the effects of weightlessness on human thermoregulation are not well understood. Specifically, studies, investigating the effects of long-duration spaceflight on CBT at rest and during exercise are clearly lacking. We here show that during exercise CBT rises higher and faster in space than on Earth. Moreover, we observed for the first time a sustained increased astronauts’ CBT also under resting conditions. This increase of about 1 °C developed gradually over 2.5 months and was associated with augmented concentrations of interleukin-1 receptor antagonist, a key anti-inflammatory protein. Since even minor increases in CBT can impair physical and cognitive performance, both findings have a considerable impact on astronauts’ health and well-being during future long-term spaceflights. Moreover, our findings also pinpoint crucial physiological challenges for spacefaring civilizations, and raise questions about the assumption of a thermoregulatory set point in humans, and our evolutionary ability to adapt to climate changes on Earth.

## Introduction

Humans are endothermic organisms with a core body temperature (CBT) of about 37 °C, which is controlled within a narrow range by the preoptic nucleus of the hypothalamus with slightly undulating circadian changes. It is well known that the tight control of CBT is a prerequisite for maintaining physical^1^ and mental^2^ performance. Severe CBT deviations (<33 °C or >40 °C) can even have life-threatening consequences^3^. Specifically, heat stress is of particular and growing concern in various occupational settings with significant social and economic impacts^4^, and it is recommended that CBT should not exceed 38.0 °C for an average worker in prolonged daily exposure to heavy work^5^. The tight regulation of CBT requires heat transfer mainly via three processes: thermal radiation, convection and evaporation^6,7^. While their relative contribution varies by workload, environmental conditions, and hydration^8,9^, any inefficiencies of these control mechanisms will result in reduced heat transfer and increase associated the risk of heat stress and hyperthermia.

During spaceflight these processes can be considerably challenged as reduced gravity impairs convective heat transfer and the efficiency of evaporation^10,11^. Such deficiencies could be particularly prominent during exercise, where >80% of energy expenditure is converted to heat. This is in line with anectodal evidence from cosmonauts complaining about thermal discomfort^10,11^, and astronauts, reporting that heat stress is a critical issue during physical exercise in weightlessness^12^. In addition, recent research suggests that spaceflight induces a pro-inflammatory response, as indicated by increases in interleukin-1 receptor antagonist (IL-1ra)^13^, a naturally occurring competitive inhibitor of interleukin 1, which has also been shown to play a crucial role in downregulating CBT^14^. While previous research has noted increases in CBT during spaceflight^15–17^ and impaired thermoregulation during space analogs, i.e. head-down tilt bed rest^18^, these studies were limited to short missions and did not investigate the effect of acute physical exercise on CBT. Given that exercise will be one of the key countermeasures of future long-duration space missions (LDSM), studies investigating the effects of thermoregulation during spaceflight are critically needed.

The primary purpose of the present study was to assess the effects of long-duration spaceflight on CBT at rest and during vigorous exercise. We hypothesized that CBT would increase more rapidly and reach a higher maximum during physical exercise during spaceflight than on Earth. In addition, we hypothesized that these changes would be paralelled by increased in IL-1ra to mitigate further increases in CBT. Clearly existing approaches to monitor deep body temperature such as rectal, pulmonary artery, distal esophagus or ingestible telemetric CBT sensors are too invasive and unsafe for long-duration CBT monitoring on space stations. Moreover, brain temperature is of particular importance given the critical role of the hypothalamus in thermoregulation. Currently only the nasopharynx, esophagus, and the pulmonary artery have been considered as reliable sites for indirectly monitoring brain temperature^19^. We therefore developed a new technology, combining a skin surface temperature sensor with a heat flux sensor, to provide a sensor that is non-invasive, sensitive enough to quantitatively reflect minor changes in arterial blood temperature, has a rapid response time and is not biased towards various environmental conditions. This approach has been tested in various space analogs and clinical settings and has been found to provide a reliable and valid estimate of CBT^20–23^. Importantly, the sensor probe can be applied to the forehead, and has been shown to be highly accurate surrogate compared to nasopharyngeal^20^, esophageal^23–27^ and artery temperatures^24^. Using this technology we tested the hypotheses of impaired thermoregulation during long-term spaceflights by investigating CBT in astronauts at rest and during exercise before, during and after six-months stays on the International Space Station (ISS).

## Results

### Temperature variables

**Figure 1:**
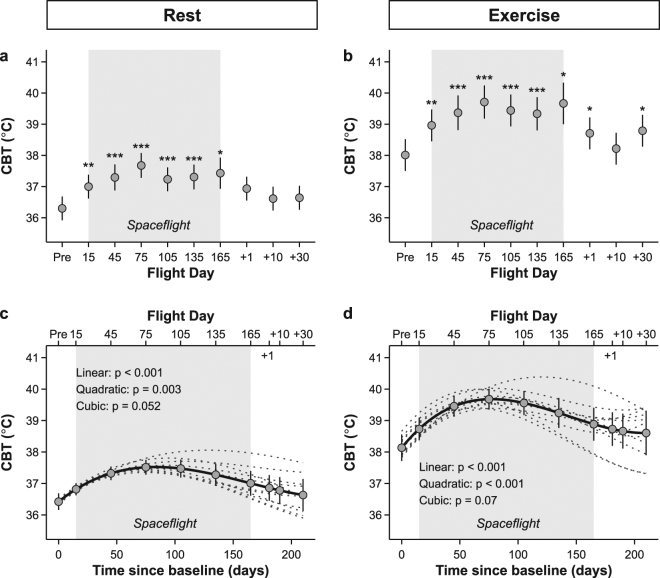
Changes in core body temperature at rest (left panel, a and c) and after exercise (right panel, b and d) during long-duration spaceflight. Grey shaded area shows time during space. Upper panels (a and b) show marginal means and 95% CI from mixed model treating time as a fixed factor. Significant levels are indicated by asterisks. Pre refers to preflight data collection. 15, 45, 75, 105, 135 and 165 indicate flight day during mission. +1, +10 and +30 correspond to the number of days when data were collected after return to Earth. Lower panels (c and d) show marginal means and 95% CI as well as individual (dotted lines) and overall (solid lines) trajectories for changes of CBT over time, resulting from mixed models treating time as a covariate (for details see methods). n = 11, missing data are detailed in Tables S1 and S2. ^***^P < 0.001, ^**^P < 0.01, ^*^P < 0.05.

**Figure 2:**
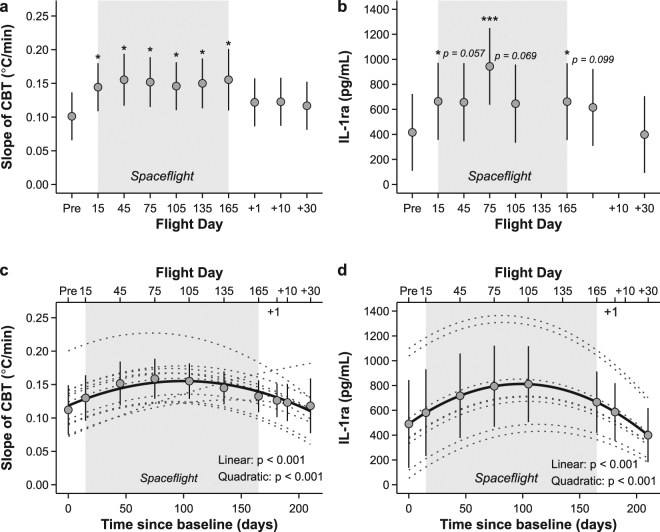
Changes of increase in core body temperature during exercise (left panel, a and c) and IL-1ra (right panel, b and d) at rest during long-duration spaceflight. Grey shaded area shows time during space. Panel a and b show marginal means and 95% CI from mixed model treating time as a fixed factor the slope of CBT during exercise and IL-1ra, respectively. Significant levels are indicated by asterisks. Pre refers to preflight data collection. 15, 45, 75, 105, 135 and 165 indicate flight day during mission. +1, +10 and +30 correspond to the number of days when data were collected after return to Earth. Panel c and d show marginal means and 95% CI as well as individual (dotted lines) and overall trajectories (solid lines) for changes of increases in CBT and IL-1ra over time, respectively, resulting from mixed models treating time as a covariate (for details see methods). Panel c shows a single subject with an unusual response, which was also confirmed by influential diagnostics (highest Cook’s D). The model was therefore rerun excluding these data, which, however, did not alter the inferential statistics, i.e. both the linear and quadratic component remained highly significant (p < 0.001). n = 11, missing data are detailed in Tables S3 and S4. ^***^P < 0.001, ^**^P < 0.01, ^*^P < 0.05.

Figure 1a and b show mean changes in CBT at rest and exercise before, during and after spaceflight. At rest CBT was increased by about 1.01 °C on the ISS compared to baseline (F (9,81) = 5.59, p < 0.001 for main effect). Similarly, maximal CBT during exercise increased significantly in space on average by 1.39 °C (F (9,81) = 6.03, p < 0.001 for main effect), and even exceeded 40 °C in some individuals during the expedition. The parameter estimates of the mixed models revealed that CBT was significantly increased throughout the space mission at each time point, reaching a plateau after 75 days of spaceflight, and only returned slowly to baseline during the recovery (Fig. 1a and b). To assess individual growth curve trajectories over time, we also performed a mixed model including time in days as a covariate. The linear, quadratic and cubic components confirmed the pattern of a gradual increase, followed by a decelerated decline in CBT (Fig. 1c and d). The slope of CBT during exercise, i.e. the rate of increaes in CBT during exercise, also increased from 0.1 °C min^−1^ on ground to 0.15 °C min^−1^ inflight (Fig. 2a). While no significant main effect was observed (F (9,81) = 1.45, p = 0.18), parameter estimates of the model indicated that after 30 days of spaceflight, the slope of CBT remained significantly elevated throughout the mission compared to baseline (Fig. 2a). In line with that, growth curve analysis showed a significant linear and quadratic component (Fig. 2b).

### Biochemical data

**Figure 3:**
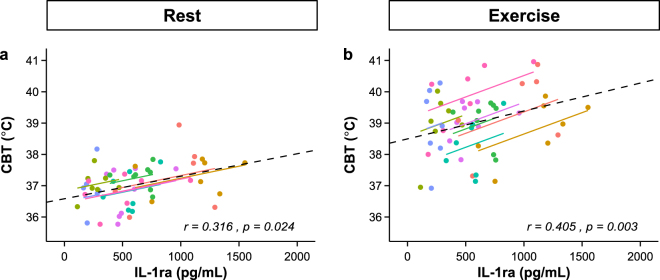
Repeated measures correlation between IL-1ra and core body temperature^46^ at rest (**a**) and after exercise (**b**) during long-duration spaceflight, to assess whether an increase in IL-1ra was associated with an increase in CBT within the individual. Dots are actual data values and grouped by subjects (each color summarizing one subject, n = 7 see also Table S4). By removing measured variance between-participants using analysis of covariance (ANCOVA), the repeated measures correlation provides the best linear fit for each participant using parallel regression lines. Solid colored lines show the repeated measures correlation model fit. Note that the availability of data points varies for individuals, reflecting different length of model fits. The multilevel model fit (dashed line) is also shown for the conditional effect (intervention, i.e. spaceflight).

IL-1ra showed a nearly identical time course as CBT (F (7,47) = 4.06, p = 0.001). As indicated in Figure 3, IL-1ra was significantly increased after 15, 75, and 165 days of spaceflight compared to baseline (p = 0.042, p < 0.001, and p = 0.044, respectively) and also followed a negative quadratic trend (F (1,53) = 18.7, p < 0.001). To assess whether an increase in IL-1ra was associated with an increase in CBT, we calculated within-subject correlations, accounting for non-independence among observations using analysis of covariance (ANCOVA) and statistically adjust for inter-individual variability. Figure 3 shows the fitted parallel lines for each subject as well as the model fit for all subjects. Both CBT at rest and maximal CBT during exercise were moderately positively correlated to IL-1ra (r(49) = 0.32, p = 0.024 and r(49) = 0.41, p = 0.003 for CBT at rest and during exercise, respectively).

## Discussion

Elevated CBT in space has been earlier observed by Gundel *et al*.^16,17^, using 24-hours CBT recordings, however to much lower extent (0.06–0.16 °C). In this case the higher resting CBT remained elevated throughout the stay on the ISS and finally returned to baseline 10 days after landing; these findings are also in line with observations of Dijk *et al*.^15^. However, these studies were short-term missions, spanning a maximum of 8–16 days of spaceflight. Our data suggest that the shift in CBT seems to be a slow, gradual phenomenon that reaches its peak only after months of spaceflight. In addition, we also observed an increase of maximal CBT after short bouts of exercise, in some cases exceeding 40 °C. Remarkably, these increases occurred despite workloads were lower inflight compared to the preflight exercise protocol^28^. Moreover, the rate of the increase in CBT during exercise, i.e. the slope of CBT, was significantly steeper during spaceflight. These findings are very much in line with earlier studies, stating that after long-term spaceflight the sensitivity of the heat-loss responses is reduced, resulting in a faster rise in CBT^10^. Furthermore, our data indicate that the impairments in thermoregulation are still prevalent after return to Earth, and recovery occurs only very gradually. In our opinion, the diminished convective and evaporative heat loss in space called for an increase in skin perfusion to enhance radiative heat loss. This could happen by marked peripheral vasodilation as recently described by Norsk *et al*.^29^, and might be an attempt of the autonomic nervous system to increase heat loss through radiation even under resting conditions in space^30^.

CBT was determined using a novel, non-invasive heat flux sensor^20,21,23,25,26^. Major advantages and limitations of the different methods including the Double Sensor, their anatomical site, their applicability in the clinic and in the field, and the role of the hypothalamus in thermoregulation, as well as the ambiguous brain temperature concepts, have been intensively reviewed and discussed in the recent literature^20,30–34^. This method allowed to monitor CBT at the forehead, accounting for important factors of brain tissue temperature and skin perfusion adjacent to the skull. Brain temperature mainly depends on heat transfer from the peripheral organs to the brain via the arterial blood and on the removal of heat from the brain via the cerebral veins. Additional heat is removed by cooler arterial blood entering the brain. Due to high metabolic activity, deep brain temperature is slightly higher than central blood temperature in the pulmonary artery (~0.2 °C) or in the esophagus (~0.3 °C) under resting conditions on Earth^33^. However, vigorous physical exercise combined with extremely high regional neuronal activity (motor and supplementary motor areas), further increases cerebral metabolic rate and as such aggravate the heat strain on the brain^35^. Caputa reports that the limit of brain temperature is 44 °C for a short period of time, or 40 to 60 minutes when in the range of 42 to 42.5 °C^36^. Since we have shown that our crew members reached CBT > 40 °C during short submaximal exercise, the safety margins for heavy exercise in space seem to be smaller. To assess the impact of any inflammatory causes on the increased CBT, we also determined IL-1ra at 15, 30, 60, 120, and 180 days of spaceflight. IL-1ra was specifically targeted as it has been shown to be particularly sensitive during spaceflight with 300- to 600-fold higher increases during spaceflight compared to IL-1α or IL-1β^13^. In fact, IL-1ra appears to play a key role in local inflammatory processes^37^ and has been shown to be more sensitive than other cytokines such as IL-1β to peripheral changes in the blood compared to alterations of specific local tissues concentrations^38^. In line with our hypothesis, IL-1ra was significantly increased during spaceflight and exhibited a nearly identical pattern to the CBT data. Moreover, we also found a moderate relationship between IL-1ra and CBT, suggesting that cytokine dysregulation is associated with increased CBT in spaceflight. The activation of these immunological pathways might have an iatrogenic origin related to the strenuous exercise programs prescribed to astronauts during long-duration space missions. While the immune system is already affected by spaceflight *per* se^39–41^, vigorous exercise induces a marked impairment of the immune system^39^. Another possible explanation for any pro-inflammatory responses during spaceflight could be related to the increased radiation exposure of astronauts in low Earth orbit^39^. According to recent measurements, the daily exposure rates measured on board the ISS have been described to be 100 times higher that exposure rates on the Earth^42^. However, despite considerable evidence for spaceflight-related cytokine dysregulation, as well as the high sensitivity of IL-1ra for plasma concentrations, it should be noted that the increases were rather moderate. In this regard, a new line of research investigating the effects of acute and chronic increases in CBT without any inflammatory causes deserves consideration. Various animal, but also an increasing number of human studies, suggest psychological stress-induced, persistent hyperthermia. These data indicate that novelty stress, such as exposure to unfamiliar environments, can increase CBT by as much as 2 °C and chronic stress can lead to the so-called stress induced hyperthermia^43^. Currently, its neural mechanisms are unknown, but are likely to include altered sympathetic activity, changes in non-shivering thermogenesis via activation of brown tissue adipose, and possible interactions between the hypothalamic–pituitary–adrenal axis, the prefrontal cortex, amygdala, orexin neurons, and the preoptic nucleus of the hypothalamus.

In summary, we found that CBT rises higher and faster during physical exercise in space than on ground, and resting CBT is elevated in long-duration spaceflight. We concluded that, within the limits of a spaceflight experiment, these increases might be related to persistent low-grade pro-inflammatory responses to weightlessness, strenuous exercise protocols, radiation, psychological stress-induced hyperthermia or a combination thereof. Irrespective of its underlying causes, this *space fever*, as we may call it, has potential implications for long-term spaceflights in terms of astronauts’ health, well-being, and support, including energy, nutrient, and fluid requirements as well as physical and cognitive performance.

## Methods

### Study Participants

Eleven astronauts (7 men, 4 women) participated in the study. Preflight anthropometric data were as follows (mean ± S.D.): age 50 ± 4 years, height 1.77 ± 0.07 m, body weight 78.5 ± 15.7 kg, body mass index 24.8 ± 3.5 kg/m^2^. The study was designed in accordance with the the Declaration of Helsinki, Revision 6, 2008. It was approved by the local Ethics Committee of the Charité Universitätsmedizin Berlin and by the Institutional Review Boards of the space agencies involved. After the purpose, procedures, and known risks had been explained, all participants provided written informed consent to particpate in the study.

### Experimental Procedures

Core body temperature was assessed at rest and during a standardized exercise protocol 90 days prior to launch (L-90 days), after 15, 45, 75, 105, 135, and 165 of spaceflight (FD (flight day)15, FD45, FD75, FD105, FD135, and FD165 (nominal duration = 180 days)), and after 1, 10 and 30 days of returning to Earth (R (recovery) + 1, R + 10, and R + 30). To assess the effect of spaceflight on IL-1ra and its association with CBT, blood samples were collected 180, 45 and 10 days before launch (L-180, L-45, and L-10), after 15, 30, 60, 120 and 180 days of spaceflight (FD15, FD30, FD60, FD120, and FD180), within 24 hours after return (R + 0) and 30 days of recovery (R + 30).

### Exercise protocol

The exercise test consisted of a submaximal protocol eliciting steady-state cardiovascular and metabolic responses. Exercise intensity was based on maximal oxygen uptake (VO_2_ max) determined during an initial preflight maximal graded exercise test taken at L-9 months. Details of this test are provided elsewhere^28^. Briefly, the protocol consisted of a 2-min resting period, followed by three continuous 5 min power levels prescribed to elicit 25%, 50%, and 75% of the individual’s preflight VO_2_ max. These were immediately followed by 1-min stepwise increments of 25 W until subjects reached their symptom-limited maximum. Data were collected at thermoneutral conditions. Baseline testing was performed at the Exercise Laboratory of the Johnson Space Center (JSC) in Houston, Texas (USA) under the following conditions: ambient temperature (Ta) 21.0 ± 1.2 °C, relative humidity 54.6 ± 13.2%, ambient pressure (Pa) 1016 ± 7 mbar, percentage of oxygen (O_2_) 20.95 ± 0.09%, percentage of carbon dioxide (CO_2_) 0.13 ± 0.07%. Testing during the space missions was conducted on the International Space Station (ISS) under the following environmental conditions: Ta 23.6 ± 1.8 °C, relative humidity 41.4 ± 3.8%, Pa 993 ± 12 mbar, O_2_ 21.7 ± 0.7%, CO_2_ 0.35 ± 0.11%. Air velocities due to artificial ventilation on the ISS are heterogeneous, i.e., for the European Columbus Module on the ISS 0.076–0.203 m/s for 67% of the module and 0.036–1.016 m/s for the entire space station^44^.

### Instrumentation and measurements

#### Core body temperature

CBT was determined using a novel heat flux sensor approach called ‘Double Sensor’ positioned at the forehead. The Double Sensor consists of two temperature probes: T1 (which is in close contact with the skin surface), and T2 (located on the top surface of the sensor). The temperature probes are separated by an insulating disc between the two thermistors. Both probes are integrated in an isolative casing. The sensor is fixed using a two-sided adhesive ring tape as well as an additional adhesive tape to securely affix it to the astronaut’s skin on the forehead. A detailed description of the sensor design and its precision, accuracy, and validity is described elsewhere^20–23,25,26^. Data were recorded and stored at 0.5 Hz using a miniaturized mobile system specifically adapted for spaceflight operations (Health Lab-System, Koralewski Industrie-Elektronik, Hambuehren, Germany). Raw CBT data were summarized using a moving average over 30 samples; i.e. recorded at 1 min intervals and separated into the following phases: pre-exercise, exercise, and post-exercise. Minimum and maximum CBT were extracted from baseline and exercise data respectively. The slope of temperature increases during exercise were obtained by linear regression analyses.

#### Interleukin-1 receptor antagonist

Given expected CBT changes inflight, we capitalized upon a parallel experiment when immunological assays were obtained on approximately the same days as the exercise trials. These whole-blood samples were collected as part of the Nutritional Status Assessment (aka Nutrition SMO)^13^. Briefly, venous blood samples (5.0 mL) were collected to determine IL-1ra, in duplicate using a commercially available multiplex bead immunoassay (R&D Systems, Minneapolis, Minnesota). The arithmetic mean of all preflight measurements was taken as the baseline.

### Data Analysis

Descriptive statistics are reported as means and standard deviations unless otherwise stated. Mixed-model analyses with subject as a random factor and time as a fixed factor were used to analyze CBT and biochemical data. Due to the small number of women (n = 4 for core body temperature and n = 2 for biochemical data), sex was not considered as a fixed factor. Covariance matrices were determined by restricted maximum likelihood (REML) estimation. For each outcome variable, two models were defined treating time as discrete factor and continuous covariate, respectively. To assess the effect of spaceflight at distinct time points, time was included as a discrete factor with random intercepts for subjects, and defining planned constrasts using the baseline measurement as a reference level^45^. To analyze individual growth trajectories, time was expressed in days with preflight data collection as baseline, i.e. 0 days, and entered as linear, quadratic, and cubic (for core body temperature only) covariates with correlated random slopes and intercepts for subjects. To reduce differences in scales, nonlinear terms were rescaled (by 1E + 3 and 1E + 05 for quadratic and cubic terms, respectively). Normality and homogeneity were checked by visual inspections of plots of residuals against fitted values. To assess associations between CBT and IL-1ra, accounting for the variation between subjects, we calculated within-subject correlations for repeated measures^46^. All statistical analyses were carried out using the software package R^47^. Mixed models were analyzed using the lme4^48^ and lmerTest^49^ package. P-values were obtained by using Satterthwaite’s approximation for denominator degrees of freedom. Within-subject correlations were determined using the rmcorr package^50^. The level of significance was set to α = 0.05 (two-sided) for all testing.

### Data Availability

The datasets generated during and/or analyzed during the current study are available from the corresponding author on reasonable request.

## Electronic supplementary material

Supplementary Material
