# Supplementary material for: Increased core body temperature in astronauts during long-duration space missions
Source: Sci Rep. 2017 Nov 23;7:16180. doi: 10.1038/s41598-017-15560-w (PMC5701078; doi:10.1038/s41598-017-15560-w)
Supplement: Supplementary file 1 — Supplementary Material [file 41598_2017_15560_MOESM1_ESM.doc]

# Supplement

# Increased core body temperature in astronauts during long-duration space missions.

Alexander C. Stahn^1,2,+^, Andreas Werner^3,+^, Oliver Opatz^1^, Martina A. Maggioni^1,4^,

Mathias Steinach^1^, Victoria Weller von Ahlefeld^1^, Alan Moore^5^, Brian E. Crucian^6^,

Scott M. Smith^6^, Sara R. Zwart^7^, Thomas Schlabs^1^, Stefan Mendt^1^, Tobias Trippel^8^,

Eberhard Koralewski^1^, Jochim Koch^9^, Alexander Choukèr^10^, Günther Reitz^11,12^,

Peng Shang^13^, Lothar Röcker^1^, Karl A. Kirsch^1^, Hanns-Christian Gunga^1,+,*^

^1^Charité – Universitätsmedizin Berlin, corporate member of Freie Universität Berlin, Humboldt-Universität zu Berlin, and Berlin Institute of Health, Institute of Physiology, Center for Space Medicine and Extreme Environments, CharitéCrossOver (CCO), Charitéplatz 1, 10117 Berlin, Germany.

^2^Division of Sleep and Chronobiology, Department of Psychiatry, Perelman School of Medicine at the University of Pennsylvania, 1019 Blockley Hall, 423 Guardian Drive, Philadelphia, PA 19104-6021, USA

^3^Zentrum für Luft- und Raumfahrtmedizin der Luftwaffe, FG I 1 – Flugphysiologisches Trainings Zentrum, Steinborner Str. 43, 01936 Königsbrück, Germany

^4^Department of Biomedical Sciences for Health, Università degli Studi di Milano, via Luigi Mangiagalli 31, 20133 Milan, Italy.

^5^Department of Health and Kinesiology, Lamar University, Beaumont, TX 77710, USA.

^6^Biomedical Research and Environmental Sciences Division, NASA Johnson Space Center, Houston, TX 77058, USA.

^7^Preventive Medicine and Community Health, University of Texas Medical Branch, 301 University Boulevard, Galveston TX 77555

^8^Charité Medizinische Klinik, Charité Universitätsmedizin Berlin, Kardiologie, Augustenburger Platz 1, 13353 Berlin, Germany.

^9^Drägerwerk AG & Co. KGaA, Moislinger Allee 53-55, 23558 Lübeck, Germany.

^10^Department of Anaesthesiology, Hospital of the University of Munich, Marchioninistrasse 15, 81377 München, Germany.

^11^DLR, Institut für Luft- und Raumfahrtmedizin, Abteilung Strahlenbiologie, Linder Höhe, 51147 Köln, Germany.

^12^Nuclear Physics Institute of the Czech Academy of Sciences, Department of Radiation Dosimetry, Na Truhlářce 39/64, 180 00 Praha 8, Czech Republic

^13^Key Laboratory for Space Bioscience & Biotechnology, Institute of Special Environnments Biophysics, School of Life Sciences, Northwestern Polytechnical University, Xi’an, 710072, China

^+^these authors contribute equally to the paper

^*^ [hanns-christian.gunga@charite.de](mailto:hanns-christian.gunga@charite.de)

| **Table S1:** Resting Core Body Temperature at Rest and During Exercise Before, During, and After Spaceflight (mean ± SD)† | | | | | | | | | | | |
| --- | --- | --- | --- | --- | --- | --- | --- | --- | --- | --- | --- |
|  | **Preflight** | **FD15** | **FD45** | **FD75** | **FD105** | **FD135** | **FD165** | **R+1** | **R+10** | **R+30** |  |
| **Men (n=7)** | | | | | | | | | | |  |
| Mean (SD) | 36.2 (±0.5) | 37.0 (±0.5) | 37.4 (±0.3) | 37.9 (±0.4) | 37.4 (±0.8) | 37.4 (±0.4) | 37.3 (±0.6) | 37.0 (±1.0) | 36.5 (±1.1) | 36.5 (±0.7) |  |
| Missing | 0 (0%) | 0 (0%) | 1 (14.3%) | 1 (14.3%) | 0 (0%) | 0 (0%) | 3 (42.9%) | 0 (0%) | 0 (0%) | 0 (0%) |  |
| **Women(n=4)** | | | | | | | | | | |  |
| Mean (SD) | 36.5 (±0.2) | 37.1 (±0.2) | 36.8 (±1.0) | 37.4 (±0.3) | 37.0 (±0.5) | 37.2 (±0.6) | 37.8 (±0.0) | 36.9 (±0.6) | 36.8 (±0.5) | 37.0 (±0.6) |  |
| Missing | 0 (0%) | 0 (0%) | 1 (25.0%) | 0 (0%) | 0 (0%) | 1 (25.0%) | 2 (50.0%) | 0 (0%) | 0 (0%) | 0 (0%) |  |
| **Total (n=11)** | | | | | | | | | | |  |
| Mean (SD) | 36.3 (±0.4) | 37.0 (±0.4) | 37.2 (±0.6) | 37.7 (±0.4) | 37.2 (±0.7) | 37.3 (±0.4) | 37.5 (±0.5) | 36.9 (±0.8) | 36.6 (±0.9) | 36.6 (±0.7) |  |
| Missing | 0 (0%) | 0 (0%) | 2 (18.2%) | 1 (9.1%) | 0 (0%) | 1 (9.1%) | 5 (45.5%) | 0 (0%) | 0 (0%) | 0 (0%) |  |
| † missing data are indicated as absolute values and percentages | | | | | | | | | | |  |

| **Table S2**: Maximum Core Body Temperature During Exercise Before, During, and After Spaceflight (mean ± SD)† | | | | | | | | | | |
| --- | --- | --- | --- | --- | --- | --- | --- | --- | --- | --- |
|  | **Preflight** | **FD15** | **FD45** | **FD75** | **FD105** | **FD135** | **FD165** | **R+1** | **R+10** | **R+30** |
| **Men (n=7)** | | | | | | | | | | |
| Mean (SD) | 38.3 (±0.7) | 38.8 (±0.7) | 39.4 (±0.5) | 39.7 (±0.5) | 39.5 (±0.7) | 39.5 (±0.7) | 39.5 (±1.1) | 38.8 (±1.2) | 38.2 (±0.8) | 38.8 (±1.2) |
| Missing | 0 (0%) | 0 (0%) | 1 (14.3%) | 1 (14.3%) | 0 (0%) | 0 (0%) | 3 (42.9%) | 0 (0%) | 0 (0%) | 0 (0%) |
| **Women(n=4)** | | | | | | | | | | |
| Mean (SD) | 37.5 (±0.5) | 39.3 (±1.1) | 39.1 (±0.5) | 39.7 (±0.9) | 39.4 (±0.7) | 38.8 (±0.9) | 39.9 (±0.1) | 38.5 (±0.9) | 38.3 (±1.0) | 38.8 (±1.4) |
| Missing | 0 (0%) | 0 (0%) | 1 (25.0%) | 0 (0%) | 0 (0%) | 1 (25.0%) | 2 (50.0%) | 0 (0%) | 0 (0%) | 0 (0%) |
| **Total (n=11)** | | | | | | | | | | |
| Mean (SD) | 38.0 (±0.8) | 39.0 (±0.9) | 39.3 (±0.5) | 39.7 (±0.6) | 39.4 (±0.7) | 39.3 (±0.8) | 39.6 (±0.9) | 38.7 (±1.1) | 38.2 (±0.8) | 38.8 (±1.2) |
| Missing | 0 (0%) | 0 (0%) | 2 (18.2%) | 1 (9.1%) | 0 (0%) | 1 (9.1%) | 5 (45.5%) | 0 (0%) | 0 (0%) | 0 (0%) |
| † missing data are indicated as absolute values and percentages | | | | | | | | | | |

| **Table S3**: Increase in Core Body Temperature During Exercise Before, During, and After Spaceflight (mean ± SD)† | | | | | | | | | | |
| --- | --- | --- | --- | --- | --- | --- | --- | --- | --- | --- |
|  | **Preflight** | **FD15** | **FD45** | **FD75** | **FD105** | **FD135** | **FD165** | **R+1** | **R+10** | **R+30** |
| **Men (n=7)** | | | | | | | | | | |
| Mean (SD) | 0.11 (±0.04) | 0.14 (±0.08) | 0.15 (±0.05) | 0.14 (±0.03) | 0.15 (±0.02) | 0.16 (±0.05) | 0.16 (±0.05) | 0.13 (±0.05) | 0.15 (±0.10) | 0.13 (±0.03) |
| Missing | 0 (0%) | 0 (0%) | 1 (14%) | 1 (14%) | 0 (0%) | 0 (0%) | 3 (43%) | 0 (0%) | 0 (0%) | 0 (0%) |
| **Women(n=4)** | | | | | | | | | | |
| Mean (SD) | 0.08 (±0.07) | 0.16 (±0.08) | 0.18 (±0.05) | 0.17 (±0.08) | 0.14 (±0.05) | 0.12 (±0.02) | 0.13 (±0.01) | 0.11 (±0.09) | 0.07 (±0.03) | 0.09 (±0.06) |
| Missing | 0 (0%) | 0 (0%) | 1 (25%) | 0 (0%) | 0 (0%) | 1 (25%) | 2 (50%) | 0 (0%) | 0 (0%) | 0 (0%) |
| **Total (n=11)** | | | | | | | | | | |
| Mean (SD) | 0.10 (±0.05) | 0.14 (±0.07) | 0.16 (±0.05) | 0.15 (±0.05) | 0.15 (±0.03) | 0.14 (±0.04) | 0.15 (±0.04) | 0.12 (±0.06) | 0.12 (±0.09) | 0.12 (±0.05) |
| Missing | 0 (0%) | 0 (0%) | 2 (18%) | 1 (9%) | 0 (0%) | 1 (9%) | 5 (45%) | 0 (0%) | 0 (0%) | 0 (0%) |
| † missing data are indicated as absolute values and percentages | | | | | | | | | | |

| **Table S4:** IL-1ra During Exercise Before, During, and After Spaceflight (mean ± SD)† | | | | | | | | | | |
| --- | --- | --- | --- | --- | --- | --- | --- | --- | --- | --- |
|  | **Preflight** | **FD15** | **FD45** | **FD75** | **FD105** | **FD135** | **FD165** | **R+1** | **R+10** | **R+30** |
| **Men (n=7)** | | | | | | | | | | |
| Mean (SD) | 488.60 (±226.87) | 673.88 (±461.91) | 458.17 (±287.08) | 999.78 (±935.14) | 651.02 (±336.37) | NaN (± NA) | 746.15 (±366.92) | 588.52 (±348.30) | NaN (± NA) | 375.88 (±118.79) |
| Missing | 3 (43%) | 3 (43%) | 4 (57%) | 3 (43%) | 3 (43%) | 7 (100%) | 3 (43%) | 3 (43%) | 7 (100%) | 3 (43%) |
| **Women(n=4)** | | | | | | | | | | |
| Mean (SD) | 343.32 (±287.63) | 654.55 (±490.91) | 688.27 (±377.24) | 886.92 (±538.47) | 746.00 (±367.81) | NaN (± NA) | 577.20 (±418.46) | 642.73 (±405.58) | NaN (± NA) | 421.22 (±204.54) |
| Missing | 0 (0%) | 0 (0%) | 0 (0%) | 0 (0%) | 1 (25%) | 4 (100%) | 0 (0%) | 0 (0%) | 4 (100%) | 0 (0%) |
| **Total (n=11)** | | | | | | | | | | |
| Mean (SD) | 415.96 (±252.08) | 664.21 (±441.40) | 589.66 (±337.27) | 943.35 (±709.00) | 691.73 (±322.87) | NaN (± NA) | 661.67 (±375.37) | 615.62 (±351.18) | NaN (± NA) | 398.55 (±156.73) |
| Missing | 3 (27%) | 3 (27%) | 4 (36%) | 3 (27%) | 4 (36%) | 11 (100%) | 3 (27%) | 3 (27%) | 11 (100%) | 3 (27%) |
| † missing data are indicated as absolute values and percentages | | | | | | | | | | |

| **Table S5:** Main Effects for Linear Mixed Model Examining the Effects of Spaceflight on Core Body Temperature and IL-1ra, Treating Time as a Factor (n=11) | | | | |
| --- | --- | --- | --- | --- |
| **Variables** | ***df1*** | ***df2*** | ***F*** | **p-value** |
| **CBT Rest** | | | | |
| Time | 9.00 | 81.41 | 5.59 | < 0.001 |
| **CBT Exercise** | | | | |
| Time | 9.00 | 81.33 | 6.03 | < 0.001 |
| **Increase in CBT During Exercise** | | | | |
| Time | 9.00 | 81.09 | 1.45 | 0.18 |
| **IL-1ra** | | | | |
| Time | 7.00 | 47.00 | 4.06 | 0.001 |
| *df1*, numerator degrees of freedom, *df2*, denominator degrees of freedom. | | | | |

| **Table S6:** Contrasts for Linear Mixed Model Using Baseline as a Reference Level (n=11) | | | |
| --- | --- | --- | --- |
| **Variables** | ***df*** | ***t*** | **p-value** |
| **CBT Rest** | | | |
| Pre | 70.07 | 189.91 | < 0.001 |
| FD15 | 80.92 | 2.87 | 0.005 |
| FD45 | 81.51 | 3.86 | < 0.001 |
| FD75 | 81.24 | 5.51 | < 0.001 |
| FD105 | 80.92 | 3.84 | < 0.001 |
| FD135 | 81.24 | 4.03 | < 0.001 |
| FD165 | 82.65 | 3.86 | < 0.001 |
| R+1 | 80.92 | 2.61 | 0.011 |
| R+10 | 80.92 | 1.29 | 0.20 |
| R+30 | 80.92 | 1.40 | 0.16 |
| **CBT Exercise** | | | |
| Pre | 67.10 | 148.87 | < 0.001 |
| FD15 | 80.86 | 2.96 | 0.004 |
| FD45 | 81.42 | 3.98 | < 0.001 |
| FD75 | 81.17 | 5.14 | < 0.001 |
| FD105 | 80.86 | 4.44 | < 0.001 |
| FD135 | 81.17 | 4.01 | < 0.001 |
| FD165 | 82.49 | 4.27 | < 0.001 |
| R+1 | 80.86 | 2.16 | 0.033 |
| R+10 | 80.86 | 0.64 | 0.52 |
| R+30 | 80.86 | 2.42 | 0.018 |
| **CBT Increase During Exercise** | | | |
| Pre | 57.66 | 5.69 | < 0.001 |
| FD15 | 80.71 | 2.00 | 0.048 |
| FD45 | 81.16 | 2.38 | 0.02 |
| FD75 | 80.95 | 2.29 | 0.025 |
| FD105 | 80.71 | 2.07 | 0.041 |
| FD135 | 80.95 | 2.21 | 0.030 |
| FD165 | 82.04 | 2.09 | 0.040 |
| R+1 | 80.71 | 0.96 | 0.34 |
| R+10 | 80.71 | 1.00 | 0.32 |
| R+30 | 80.71 | 0.73 | 0.47 |
| **IL-1ra** | | | |
| Pre | 13.85 | 2.91 | 0.012 |
| FD15 | 47.13 | 2.09 | 0.042 |
| FD45 | 47.20 | 1.95 | 0.057 |
| FD75 | 47.13 | 4.44 | < 0.001 |
| FD105 | 47.20 | 1.86 | 0.069 |
| FD165 | 47.13 | 2.07 | 0.044 |
| R+1 | 47.13 | 1.68 | 0.099 |
| R+30 | 47.13 | -0.15 | 0.88 |
| *df*, degrees of freedom | | | |

| **Table S7**: Main Effects for Linear Mixed Model Examining the Effects of Spaceflight on Core Body Temperature and IL-1ra, Treating Time as a Covariate (n=11) | | | | |
| --- | --- | --- | --- | --- |
| **Variables** | ***df1*** | ***df2*** | ***F*** | **p-value** |
| **CBT Rest** | | | | |
| Time Linear | 1.00 | 77.25 | 21.73 | < 0.001 |
| Time Quadratic | 1.00 | 81.51 | 9.63 | 0.003 |
| Time Cubic | 1.00 | 71.77 | 3.91 | 0.052 |
| **CBT Exercise** | | | | |
| Time Linear | 1.00 | 69.02 | 30.72 | < 0.001 |
| Time Quadratic | 1.00 | 77.45 | 15.69 | < 0.001 |
| Time Cubic | 1.00 | 59.35 | 7.86 | 0.007 |
| **Increase in CBT During Exercise** | | | | |
| Time Linear | 1.00 | 78.39 | 9.10 | 0.003 |
| Time Quadratic | 1.00 | 85.68 | 9.54 | 0.003 |
| **IL-1ra** | | | | |
| Time Linear | 1.00 | 51.89 | 16.43 | < 0.001 |
| Time Quadratic | 1.00 | 53.94 | 18.66 | < 0.001 |
| *df1*, numerator degrees of freedom, *df2*, denominator degrees of freedom. | | | | |
